# Residents’ satisfaction with primary medical and health services in Western China

**DOI:** 10.1186/s12913-017-2200-9

**Published:** 2017-04-21

**Authors:** Weinan Dong, Qingyu Zhang, Chunsheng Yan, Wanling Fu, Linlin Xu

**Affiliations:** 1 School of Public Health, Lanzhou University, Chengguan district, Road Dingxinan 23, Lanzhou, Gansu Province 730000 China; 2 ᅟ, Hogbin Dr, Coffs Harbour, NSW2450 Australia; 3 Tuanjiexincun Community health service centre, Chengguan district, Gansu Province 730000 China

## Abstract

**Background:**

Currently, China is in the process of medical and health care reform, and the establishment of primary medical and health services covering urban and rural residents is an important aspect of this process. Studying the satisfaction of residents of underdeveloped areas with their primary medical and health services and identifying the factors that can increase the satisfaction of different groups may improve patient compliance and ultimately improve health. Moreover, such research may provide a reference for the development of medical and health undertakings in similarly underdeveloped areas.

**Methods:**

A face-to-face survey was conducted on a stratified random sample of 2200 residents in Gansu by using structured questionnaires. Demographic characteristics were collated, and questionnaires were factor-analysed and weighted using SPSS software to obtain scores for each factor, as well as total satisfaction scores. The characteristics of poorly satisfied populations were determined by a multiple linear regression analysis using SAS software. A cluster analysis was performed using SAS software for classification and a separate discussion of populations.

**Results:**

The hypertension self-awareness rate (11.29%) of the sampled population was lower than the average hypertension prevalence (23.85%), as recorded in the 2014 Health Statistical Yearbook of the region. The disease knowledge awareness factor was the lowest factor (2.857), whereas the policy awareness factor was the highest factor (4.772). The overall satisfaction was moderate (3.898).

The multivariate linear regression model was significant (*p* <0.05). The regression coefficients were -0.041 for minors; 0.065 for unemployed people; and 0.094 for people with an elementary school educational level, a value lower than that of other population groups.

A cluster analysis was used to divide the respondents into five groups. The overall satisfaction was lowest in the second population group (rural, middle-aged)(Fz = 3.64) and was highest in the fourth population group(minors) (Fz = 4.13). Different population groups showed different satisfaction rates in F1 to F6.

**Conclusion:**

Hypertensive patients had low self-awareness, and residents had a poor grasp of disease and limited health knowledge. Their overall satisfaction was moderate. Residents expressed comparatively high satisfaction with the current policy. Minors, adults with low level of education, unemployed people and other vulnerable groups expressed low overall satisfaction. The degree of satisfaction varied greatly among the different groups. Targeted medical and health practices should be implemented for different groups; additionally, the public health practice should be strengthened.

**Electronic supplementary material:**

The online version of this article (doi:10.1186/s12913-017-2200-9) contains supplementary material, which is available to authorized users.

## Background

Since the WHO introduced the Primary Health Care (PHC) strategy in the Alma-Ata Declaration of 1978, various countries have become committed to the development of health care. Primary Health Care is the basic health care that can generally be obtained by individuals and families in a community. The Declaration stated that the health inequalities between developed and developing countries and within countries should be of concern to all countries and that health care for all and a reduction of the gap in health status between developing and developed countries were the primary goals [1]. Thirty years later, the WHO and member states reaffirmed the importance of the PHC strategy [2, 3] and emphasized the broad involvement of social factors, as well as the roles and functions of government in the health field [4]. In those three decades, the PHC strategy has played a significant role, especially in low- and middle-income countries [5].

In 1988, the Chinese government made a commitment to this global health strategy and has since been devoted to medical and health care reform [6]. Medical and health undertakings have developed tremendously, and the level of medical care has continually improved, thereby providing better medical and health services for all people in the country [7]. With the improvement of the overall level of medical care, medical sub-specialties have been further divided, and general practitioners able to meet the primary care demands have become increasingly lacking, thus indirectly leading to difficulties and higher costs associated with obtaining medical services [8]. From 1998 to 2013, the Chinese government conducted a total of five national health services surveys, which have revealed a continuously growing demand for health services in China and the disease spectrum gradually shifted towards chronic diseases. According to the fourth survey, conducted in 2008, health service utilization in China reached a record high; and according to the fifth survey, conducted after the new health reform, residents’ confidence in medical staff had increased. The difficulties and high costs of obtaining medical services have improved [9–11], but nevertheless, these two issues remain the most direct and practical concern of residents, especially for patients with common and frequently occurring diseases that need to be resolved in primary medical and health care institutions. Under the new health reform policy, the health resources shift towards the primary level, and primary medical and health services become the focus of medical and health work, which are considered to be key to solving the problem of medical services that are difficult to obtain and expensive [12, 13]. Only by constantly improving the capabilities of primary medical and health services and gradually enhancing patients’ satisfaction can residents’ health levels be improved.

### Literature review

Assessment of residents’ satisfaction with primary medical and health services is a complex process. As early as the mid-20th century, many precedents had already been established for assessing health services on the basis of patients’ opinions and comments [14]. Satisfaction is often used to evaluate the health services received by patients in health institutions [15–18], and it is influenced by numerous factors such as personal health status, demographic characteristics, socio-psychological factors, and characteristics of the health system. The improvement of the patient satisfaction can promote compliance and improve health [19]. Measurement of the patient satisfaction also involves different indicators from multiple dimensions, such as satisfaction with the daily care received, satisfaction with the information provided, and satisfaction with the healthcare environment [20], but there is not yet a gold standard method or indicator for assessing patient satisfaction [21–25].

Li Jian-Jun and others have summarized the research on satisfaction published before 2000 and have found that interview methods (telephone and face-to-face)generate higher responses than mail surveys, and only 20% of studies consider the potential importance of expectations [26]. Sara N Bleich and Emre Ozaltinb, in a 2009 study, have noted that participant satisfaction is critically influenced by their expectations [27]. Some other studies have reached the same conclusion [28–31].

A study by Zhijian Li and colleagues has evaluated residents’ satisfaction with community health services after health care system reform in Shanghai, China. The authors have found that satisfaction across all dimensions improved since the reform was initiated, but disadvantaged groups had overall poorer satisfaction levels [32]. A study by Sarah Atkinson and Dave Haran in one of Brazil’s poorest states, has found that user satisfaction can be a useful management tool. It was also found that the results of satisfaction assessment are influenced by the definition used and the method(s) used to measure satisfaction, so exploration from multiple perspectives is helpful in explaining the complexity of satisfaction [33].

In this paper, the methods and indicators used to assess the residents’ satisfaction with primary medical and health services were selected after analysis of numerous references and the results of China National Health Services Surveys, which cover public health, chronic diseases, infectious diseases and other aspects of healthcare. The assessment concentrates specifically on residents’ satisfaction with primary medical and health services under the new health reform in China.

Furthermore, the levels of satisfaction vary greatly among different countries and districts, owing to racial or ethnic and socio-economic factors [34]. The area investigated in this paper was Gansu, which is located in the northwest of China and is an economically disadvantaged region of the country, with slow development of medical and health services [35] and a large rural population. In 2014, the total GDP of Gansu ranked fourth in China, but the per capita GDP ranked last in the country, largely because the economic structure is agriculture-based, with a few large concentrated industrial bases. Additionally, Gansu, which is in the process of urbanization, has typical features of developing countries.

Studying the satisfaction of residents of underdeveloped areas with their primary medical and health services and identifying the factors that can increase the satisfaction of different groups may improve patient compliance and ultimately improve health. Moreover, such research may provide a reference for the development of medical and health undertakings in similarly underdeveloped areas.

There is extensive literature regarding the satisfaction of residents and patients. Several studies have investigated residents’ satisfaction in less-developed areas under health care system reform. However, few studies have assessed patient satisfaction from multiple objective and subjective perspectives by using multiple linear regression with dummy variables, and studies focused on the various aspects of vulnerable members’ satisfaction have been especially rare.

## Methods

### Sampling

According to the Gansu Provincial Health Department’s 2014 annual assessment of primary medical and health services, five counties/districts in Gansu (Chengguan District of Lanzhou, Gaolan County of Lanzhou, Zhuanglang County of Pingliang, Liangzhou District of Wuwei, Ganzhou County of Zhangye) were selected for this study after comprehensive consideration of their economic development status, health work foundation, and the ethnic and geographical features throughout the province. In each sampled county/district, three communities/villages were randomly selected, and the number of questionnaires was determined on the basis of the population of each community/village. A randomized household survey by trained investigators was used. The inclusion criteria for the participants were residents > 9 years of age served by the local healthcare service providers. The exclusion criteria were residents who refused to provide informed consent and residents who were unable to answer the questionnaire (for psychiatric reasons). A total of 2200 questionnaires were distributed, and 2038 valid questionnaires were returned, with a response rate of 92.64%.

### Questionnaire

To avoid variations due to the questionnaire, the questionnaire was designed after examination of a large number of related studies and was improved according to pre-survey results. The questionnaire was divided into two parts: basic information about the residents and survey contents. The basic information included sex, age, occupation, household registration, work, educational level and prevalence of diabetes and hypertension, and the survey content involved knowledge of national public health programs, health knowledge, satisfaction with primary medical and health services and health policy, which was expected to comprehensively analyse residents’ satisfaction with both objective and subjective dimensions Additional file 1.

In this paper, the alpha reliability coefficient method was used to test the reliability of the questionnaire [36]. The main consideration was whether there was high intrinsic consistency across the questionnaire items or indicators. The results of the analysis revealed high intrinsic consistency, because the inter-item reliability coefficient was 0.781, close to 0.8. Therefore, the questionnaire results were credible.

The survey used a sub-questionnaire from the third-party evaluation projects of the Gansu National Public Health Equalization Program; the sub-questionnaire has been revised by several experts and professors after extensive analysis of references and has been applied in various projects for many years. As a result, the validity of the questionnaire was high.

### Statistical analysis

After collecting the questionnaires, data were entered by double-entry using EPIDATA.

- Data were organized in EXCEL, the demographic characteristics were descriptive analyzed in order to comprehensively understand the distribution Additional file 2.
- Because the main part of the questionnaire involved many questions, a factor analysis was performed on 26 questions in the main questionnaire using SPSS 20.0 software to reduce dimensionality. According to the weights, scores 1 ~ 5 and total score was calculated. The purpose is Standardization and visualization of the satisfaction.
- A multiple linear regression analysis was used to explore the influence of each feature on the total score Fz in SAS 9.4.
- In order to further distinguish the six factor scores of each group,various characteristic values and factors were classified by a two-step cluster analysis using SAS 9.4 software, and then discuss the satisfaction in each group.
- Substantial amounts of data would have been lost if an individual case deletion method was adopted when values were missing, owing to the large number of indicators in the main survey. Therefore, the missing data in the main survey were substituted by mean imputation. When data were missing from the basic information, the case was deleted to ensure accuracy.

### Limitations of the study

There are several limitations of this study. First, we were unable to cover the entire province, owing to the limited personnel available. Second, face-to-face research was assisted by the staff members from the health services institution, which may have influenced the responses regarding satisfaction. Third, the sample size was too small to allow the participants to be divided into different groups when the data were analysed.

## Results

### Descriptive analysis

From the basic information portion of the questionnaire, the demographic characteristics and the sources of health knowledge (from multiple choice question) for the respondents are summarized in Table 1

**Table 1:** Distribution of respondents and the health knowledge sources

Sample characteristic	Level	Number of respondents (n)	Percentage (%)
Sex	Male	1084	53.19
	Female	954	46.81
Age	9~	329	16.14
	18~	451	22.13
	31~	423	20.76
	41~	394	19.33
	51~	298	14.62
	65~	138	6.77
	Blank	5	0.25
Region	Lanzhou Chengguan	468	22.96
	Lanzhou Gaolan	289	14.18
	Pingliang	506	24.83
	Wuwei	352	17.27
	Zhangye	423	20.76
Registration	Urban	826	40.53
	Rural	1161	56.97
	Alien permanent	32	1.57
	Blank	19	0.93
Occupation	Civil servant/enterprise and public institution/technician	362	17.76
	Worker/migrant labourer/farmer	749	36.75
	Retired/student	627	30.77
	Unemployed/self-employed	179	8.78
	Others	115	5.64
	Blank	6	0.29
Education	Elementary school	373	18.30
	Junior high school	586	28.75
	Senior high school	628	30.81
	Bachelor’s	429	21.05
	Master’s	12	0.59
	Blank	10	0.49
Do you have hypertension	Yes	230	11.29
	No	1802	88.42
	Blank	6	0.29
Do you have diabetes	Yes	77	3.78
	No	1954	95.88
	Blank	7	0.34
Health knowledge sources	Media	1777	87.19
	Health information column	642	31.50
	Community information column	681	33.42
	Home health workers	404	19.82
	pamphlets	630	30.91
	Doctors	375	18.40
	Residents	530	26.01
	Others	120	5.89
Total		2038	100.00

The prevalence of hypertension in the patient sample was 11.29%. According to the provincial statistical department’s documents, the prevalence of hypertension among Gansu’s residents was 23.85%.

### Analysis of factors influencing residents’ satisfaction

Because the main part of the questionnaire involved many questions, a factor analysis was performed on 26 questions in the main questionnaire using SPSS 20.0 software to reduce dimensionality. The KMO coefficient, a measure of sampling adequacy, was 0.862, which was greater than 0.5. The approximate χ2 in Bartlett’s test was 14288.411, with 325° of freedom and *p* = 0.000, thus indicating that the 26 questions selected were suitable for the factor analysis.

We assessed the variance contributions of various factors, and those with eigenvalues greater than 1 were extracted. Kaiser standardized orthogonal rotation was performed, which converged after six iterations. On the basis of the component matrix after rotation, six factors were extracted.

The answers to each questionnaire question were rated on a Likert scale: very satisfied-5, moderately satisfied-3, and very dissatisfied-1. Awareness objective questions were scored as follows: correct-5, incorrect-1. The higher the score, the higher the respondents’ objective and subjective satisfaction, and vice versa. Six factors and questions for each factor were weighted using the Delphi method [37] to obtain scores for six factors F1, F2, F3, F4, F5, F6, as well as overall score Fz. The details of the six factors and the distribution of various factors are shown in Table 2.

**Table 2:** Details of the six factors and the scores of various factors

Six factors	Details of each factor	mean	SD
Subjective satisfaction factor F1	Satisfaction with service attitude	3.42	0.9
	Satisfaction with professional skills		
	Satisfaction with medical facilities		
	Satisfaction with therapeutic effects		
	Satisfaction with institutional environment		
	Satisfaction with convenience		
	Satisfaction with disease description		
	Satisfaction with waiting time		
	Satisfaction with drug prices		
	Satisfaction with privacy protection		
Public health programme awareness factor F2	National primary public health services	3.76	1.19
	Health records for everyone		
	Health examination for elderly aged 65+		
	Management of severe mental illness		
	Health institutions should distribute health education materials		
	Health institutions provide chronic disease check-ups for people aged 35+		
	Maternal visits by health institutions		
Disease knowledge awareness factor F3	How many times should one get hepatitis B vaccinations	2.86	1.22
	HIV transmission routes		
	TB transmission routes		
Policy awareness factor F4	Importance of policy	4.77	0.75
	Necessity of policy continuation		
Dietary health awareness factor F5	Cooking oil 25 ml	4.28	1.19
	Cooking salt 6 g		
Healthy lifestyle awareness factor F6	Obesity leads to other diseases	4.6	0.92
	Hot foods should not be taken for long period		
Overall score Fz	(5*F1 + 1*F2 + 2*F3 + 3*F4 + 2*F5 + 2*F6)/(5 + 1 + 2 + 3 + 2 + 2)	3.9	0.52

The residents’ overall satisfaction was 3.90, which was moderate. The score for disease knowledge awareness was the lowest factor, at only 2.86. The residents’ subjective satisfaction with primary medical and health services was low at 3.42. The health policy awareness score was the highest factor; that is, residents were very satisfied with the primary medical and health policies.

### Multiple linear regression

A multiple linear regression analysis was used to explore the influence of each feature on the total score Fz. The overall satisfaction score Fz was set as the dependent variable, and the age group, household registration, education and occupation were set as independent variables, but the independent variables were polytomous variables. In most of the recent satisfaction studies, the independent variables have been considered polytomous variables in the logistic regression analysis, which has failed to reflect the differences in satisfaction among various feature subgroups. Therefore, in this paper, n-1 dummy variables were introduced for each independent variable, where n was the number of categories in the polytomous variables, and the dummy variables were (0,1) variables. The purpose of introducing dummy variables was to quantify the unquantifiable variables. The value of the dummy variables was usually 0 or 1. The introduction of dummy variables made the linear regression model more complex, but the problem description became more concise, and the satisfaction of different subgroups within each group was visible, which was closer to reality.

To simplify the analysis, the last item in each polytomous variable was set as the control dummy variable; i.e., the elderly subgroup in the age group;the alien permanent population in the household registration; master’s degree or above in the educational level; and other occupations in the occupation category. Because the dummy variables in one element were an indivisible whole, they had to be entered at the same time in one block during variable screening. Stepwise variable selection, with dummy variables simultaneously entered in one block, was set for the independent variables and Fz was the dependent variable. The results showed that the model could be used to analyse and predict the findings (*p* = 0.000 < 0.05), as shown in Tables 3.

**Table 3:** Linear regression parameter estimation and testing

Variable	Parameter Estimate	Pr > t
9 ~ (X1)	−0.041	0.469
18 ~ (X2)	0.018	0.349
31 ~ (X3)	0.036	0.492
41 ~ (X4)	−0.017	0.737
51 ~ (X5)	0.035	0.493
Civil servant/enterprise and public institution/technician (X6)	0.244	0.000
Worker/migrant labourer/farmer (X7)	0.189	0.000
Retired/student (X8)	0.080	0.136
Unemployed/self-employed (X9)	0.065	0.285
Elementary school(X10)	0.094	0.535
Junior high school (X11)	0.233	0.120
Senior high school (X12)	0.114	0.443
Bachelor’s (X13)	0.153	0.299
Urban (X14)	0.100	0.272
Rural(X15)	0.189	0.038
Sex	excluded from the model	
Hypertension	excluded from the model	
Diabetes	excluded from the model	

The regression coefficient referred to the disparity between the dummy variable group and control groups. Because p was largely influenced by the sample size, and variables were entered into the model gradually, the regression equation in case the model was significant (*p* <0.05 in Table 4.1) could be obtained as Yz = 3.461-0.041*X1 + 0.018*X2 + 0.036*X3 + …… + 0.189*X15. Positive and negative regression coefficients indicated positive and negative correlations. The greater the absolute value, the higher the degree of influence.

**Table 4:** The results of the two-step cluster analysis

	Category 1	Category 2	Category 3	Category 4	Category 5
Number of respondents (n)	391	316	432	441	431
Percentage (%)	19.4	15.7	21.5	21.9	21.4
Age	18–30	Minors	Middle-aged, mostly 30–50	Middle-aged, mostly 30–50	Elderly
Education	Mostly senior high school or bachelor’s degree	High school	Mostly senior high school or bachelor’s degree	Low levels of education	Low levels of education
Occupation	Mostly government, enterprises and public institutions	Students	Mostly government, enterprises and public institutions	Mostly workers and farmers	Retired
Registration	Both	Both	Mostly urban	Mostly rural	Both
Disease awareness	A few know they have hypertension or diabetes	A few know they have hypertension or diabetes	A few know they have hypertension or diabetes	A few know they have hypertension or diabetes	Some know they have hypertension or diabetes

According to the regression coefficients of the regression equation, the influence of each polytomous variable on the overall satisfaction could be obtained, i.e., the influence of the presence or absence of the dummy variable on Yz. The PE of the control dummy variables in each term was 0.

### Cluster analysis

Two-step clustering analysis uses BIRCH(balance Iterative Reducing and Clustering using Hierarchies, which is focus on the large sample clustering in hierarchical clustering. This algorithm summarizes the information of cluster through the clustering characteristics, and then cluster. Centroid distance between two cluster is indicated by Euclidean Distance and Manhattan Distance. The distance between each sample point in two cluster is indicated by average inter-cluster distance, average intra-cluster distance and variance increase distance. In the first step, the measurement of changes in distances is logarithm Likelihood function. In the second step, the result in step 1 is involved in, and each sample is cluster analyzed.

Using SAS 9.4 software, various characteristic values and factors were classified by a two-step cluster analysis, which yielded good results. The 2038 respondents were divided into five groups after individual case exclusion as shown in Table 4.

The mean factor scores for the above five groups of people are shown in Table 5.

**Table 5:** The factor scores for the five groups

	Category 1	Category 2	Category 3	Category 4	Category 5
F1	3.21	3.39	3.12	3.68	3.66
F2	4.06	3.21	3.81	4.17	3.43
F3	3.01	2.71	3.28	3.24	2.01
F4	4.85	4.38	4.81	4.83	4.91
F5	4.15	3.79	4.51	4.52	4.29
F6	4.54	4.26	4.86	4.74	4.49
Fz	3.88	3.64	3.87	4.13	3.90

As can be clearly seen from the results, for the overall satisfaction Fz, the fourth category of people (rural, middle-aged) had the highest score (Fz = 4.13), the second category (minors) had the lowest score (Fz = 3.64), and the other three groups exhibited almost no differences. The middle-aged population stood out among groups regarding dietary health knowledge and healthy lifestyle knowledge (F5 = 4.51, 4.52; F6 = 4.86, 4.74), thus indicating that the middle-aged were more concerned about health knowledge and received better health education. For F1, the subjective satisfaction with primary medical and health services, the middle-aged rural population and elderly population scored higher (F1 = 3.68, 3.66), whereas the middle-aged urban population scored lower (F1 = 3.12). The elderly group scored low in factors related to the public health programme and disease knowledge awareness (F2 = 3.43, F3 = 2.01), but they expressed high subjective satisfaction with the medical and health services. The minors had a low awareness of health policies and low levels of various health knowledge (F2, F4, F5, F6).

## Discussion

- The prevalence of hypertension in the sample, i.e., the hypertension self-awareness rate of the respondents (11.29%), was much lower than the average prevalence of hypertension,as recorded in the 2014 Gansu Health Statistical Yearbook (23.85%). The awareness rate of hypertensive patients was 47.34%. Because the question of whether the respondents had hypertension was filled in by the respondents themselves, some hypertensive patients were unaware of whether they had hypertension. In Wang Huicheng et al.’s survey in 2011, the awareness rate of hypertensive patients was also below 50% [38]. The reasons for this may include the following: 1. primary medical and health institutions’ screening for chronic conditions was inadequate; 2. residents lacked health knowledge and were not sufficiently aware of chronic diseases. The results of the China National Health Services Surveys showed that chronic diseases have become the biggest health problem for Chinese residents [9–11]. Thus, it is imperative to enhance the screening and management of chronic diseases in primary medical and health institutions.
- Table 1 shows that the number of residents who gained health knowledge via the media was much higher than the numbers of those using other means of obtaining knowledge (87.19%). The effect of health education via primary medical and health institutions was moderate (information columns from health institutions, 31.50%; community information columns, 33.42%; information pamphlets, 30.91%). Health knowledge disseminated by health professionals (home health workers, 19.82%; doctors, 18.40%) was not a common source of knowledge. Clearly, medical and health practitioners’ dissemination of health knowledge during their work did not yield any obvious effects. The purpose of health communication is to improve the people’s health level [39–41]. The media and modes of communication are also continuously updated [42]. Research on organizational health communication and “doctor-patient relationship”-centred interpersonal health communication are important topics in the field of health communication [43]. A previous study has found that patients with higher satisfaction have higher trust in doctors, are more prone to follow doctors’ advice and are more responsible for their own health [44]. Therefore, enhancement of satisfaction will actively promote health communication. Health communication, in contrast, aims to improve the public health level, which is another way in which satisfaction influences universal health. Health workers play a vital rolein both of these processes. According to the results of the Fifth China National Health Services Survey, doctor-patient relationships are gradually improving in China [11]. Therefore, in further reform, the health workers’ roles in popularizing health knowledge should be emphasized, focus should be placed on improving the professionalism and enthusiasm of primary health workers, and relevant incentives should be established.
- As seen in Table 2, the residents’ overall satisfaction was moderate (Fz = 3.898), and the subjective satisfaction with primary medical and health institutions was not high (F1 = 3.42);the grasp of disease knowledge was poor (F3 = 2.857), but the satisfaction with national health policy was high (F4 = 4.772). Bernhart and some others have claimed that patient satisfaction in underdeveloped areas is mainly associated with the therapeutic effect, i.e., whether or not the diseases were cured [45]. Improving the service capabilities of primary medical and health institutions is important for enhancing residents’ satisfaction; however, health communication and health education cannot be ignored. Policy implementation is particularly important when residents have high satisfaction with the national policies. Translating research findings to health policy is a complex process. Government’s leadership and supervision in the reform and coordination of various departments are important elements for policy implementation and overcoming the impediment of stakeholder factors during the implementation has a positive impact on the progress of health care reform [46].
- Minors (−0.041), unemployed people (0.0650) and people with an elementary school education level (0.094) exhibited regression coefficients lower than those of other populations within the same groups. The clustering results showed that elders had the poorest awareness of disease (F3 = 2.01), thus suggesting that satisfaction of vulnerable groups should be given greater attention. Zhijian Li1, JialeHou et al. have also highlighted the issue of poor satisfaction of vulnerable groups in their study performed in Shanghai, China in 2011. Vulnerable groups are less able to pay and are unable to control health care costs if health institutions are profit-motivated [32]. In future healthcare reform, medical undertakings should be focused on public welfare, and the reform of the medical reimbursement system should improve the status of vulnerable groups. In addition, health institutions should provide minors, less-educated people, the elderly, the unemployed and other vulnerable groups with targeted health services and health information.
- The clustering results showed that the middle-aged population’s grasp of health knowledge was broad and optimistic and that middle-aged rural subjects had high overall satisfaction. This finding was consistent with the results of the Fifth National Health Services Survey and the results of the study by Sarah Atkinson in Brazil [11, 33]. However, the middle-aged urban population had the lowest subjective satisfaction with primary medical and health services, perhaps because this group has higher health literacy, more affluent living conditions and higher demands for health services. Therefore, primary health services cannot meet their sophisticated health needs. The literature has demonstrated that the expectations for medical and health services affect the satisfaction of patients [47]. Elderly subjects expressed high subjective satisfaction with the current primary health services (F1 = 3.66) but lacked health knowledge. The reason for this may be that they experienced poor health services in the early years of new China or even earlier, so their expectations for health services are low. Primary medical and health institutions should explain health knowledge more patiently and meticulously to the elderly.

Because the expectations and demands for health services may affect the satisfaction of residents, concrete implementation of graded medical care is crucial to improve residents’ satisfaction while avoiding unnecessary waste of medical resources. During the Eighteenth Session of the Standing Committee of the Twelfth National People’s Congress held on December 22, 2015, the Chinese government proposed a health reform plan for accelerating the construction of a graded medical care system [48]. The progress of this graded medical care system will be examined in further studies.

- China is in a new stage of health care reform at present, wherein institutional improvement and health resource reallocation are under way. In this paper, underdeveloped areas were selected for study. Subjects were grouped by cluster analysis, and a multiple linear regression was performed introducing dummy variables. Various vulnerable groups with low satisfaction were analysed, and targeted policy-based suggestions were presented. There have been very few similar studies published in the past.
- In the next phase of research, we will increase the sample size and control the number of dummy variables entered into the multiple linear regression to obtain more refined results. In addition, we will track the changes in vulnerable groups’ satisfaction during the reform, as well as the progress of the implementation of a graded medical care system.

## Conclusion

In general, hypertensive patients had low self-awareness, and residents had a poor grasp of disease and health knowledge. The overall satisfaction level was moderate. Residents expressed comparatively high satisfaction with policy. Minors, adults with low levels of education, unemployed people and other vulnerable groups expressed low overall satisfaction. The degree of satisfaction varied greatly among different groups. Targeted medical and health practices should be implemented for different groups, and public health practice should be strengthened.

## Additional files

Additional file 1: Questionnaire about residents’ satisfaction with primary medical and health services in Gansu Province. This questionnaire is about the satisfaction of residents, which is the way to collect the data in the research. (DOCX 16 kb)

Additional file 2: Data about residents’ satisfaction in Gansu Province. This raw data was imported with epidata without any management. (XLSX 526 kb)
