# Supplementary material for: Residents’ satisfaction with primary medical and health services in Western China
Source: BMC Health Serv Res. 2017 Apr 21;17:298. doi: 10.1186/s12913-017-2200-9 (PMC5399818; doi:10.1186/s12913-017-2200-9)
Supplement: Supplementary file 1 — Questionnaire about residents’ satisfaction with primary medical and health services in Gansu Province. This questionnaire is about the satisfaction of residents, which is the way to collect the data in the research. (DOCX 16 kb) [file 12913_2017_2200_MOESM1_ESM.docx]

Questionnaire about residents’ satisfaction with primary medical and health services in Gansu Province

Site: city district/county village/community

1.Demographic information

- 1. Gender: ①male②female birth: XXXX/XX

household type: ①town ②rural area ③migrant residents

- 1. Special groups( maybe multiple-choice): ①elder（≥65）②hypertension③DM ④the maternal ⑤children guardian

1.3 Education: ①Elementary school or below②junior high school③senior high school/Secondary specialized school ④university/college ⑤master degree or above

1.4 Occupation: ①civil servant/enterprise and public institution/technician②worker/migrant worker③labourer/farmer ④retired/student ⑤unemployed/self-employed

2. Public health programme

2.1 Do you know National primary public health services : ①Y ②N

2.2 Do you know Health records ought to be for everyone: ①Y ②N

2.3 Do you know Health examination for elderly aged 65+ : ①Y ②N

2.4 Do you know Management of severe mental illness: ①Y ②N

2.5 Do you know Health institutions should distribute health education materials : ①Y ②N

2.6 Do you know Health institutions provide chronic disease check-ups for people aged 35+ : ①Y ②N

2.7 Do you know Maternal visits by health institutions: ①Y ②N

3. health knowledge:

3.1 How many times should one get hepatitis B vaccinations:

3.2 HIV transmission routes:

3.3 TB transmission routes:

3.4 Cooking oil should be less than 25ml per day: T or F

3.5 Cooking salt should be less than 6g per day: T or F

3.6 Obesity leads to other diseases: T or F

3.7 Hot foods should not be taken for long period: T or F

3.8 Health knowledge sources: ①media②Health information column ③Community information column ④Home health workers ⑤pamphlets ⑥Doctors ⑦Residents ⑧Others

4. Service satisfaction:

4.1 service attitude ①good ②modest ③poor ④hard to evaluate

4.2 professional skills ①good ②modest ③poor ④hard to evaluate

4.3 medical facilities ①good ②modest ③poor ④hard to evaluate

4.4 therapeutic effects ①good ②modest ③poor ④hard to evaluate

4.5 institutional environment ①good ②modest ③poor ④hard to evaluate

4.6 convenience ①good ②modest ③poor ④hard to evaluate

4.7 disease description ①good ②modest ③poor ④hard to evaluate

4.8 waiting time ①good ②modest ③poor ④hard to evaluate

4.9 drug prices ①good ②modest ③poor ④hard to evaluate

4.10 privacy protection ①good ②modest ③poor ④hard to evaluate

5. About the policy

5.1 National primary public health services policy is of vital importance to residents’ health. ①Y ②N

5.2 It is necessary to continue the policy. ①Y ②N
